# Diversity of *Aplochiton* Fishes (Galaxiidea) and the Taxonomic Resurrection of *A. marinus*

**DOI:** 10.1371/journal.pone.0071577

**Published:** 2013-08-19

**Authors:** Dominique Alò, Cristián Correa, Carlos Arias, Leyla Cárdenas

**Affiliations:** 1 Instituto de Conservación, Biodiversidad and Territorio, Facultad de Ciencias Forestales y Recursos Naturales, Universidad Austral de Chile, Valdivia, Chile; 2 Department of Biology and Redpath Museum, McGill University, Montreal, Quebec, Canada; 3 Smithsonian Tropical Research Institute, Panamá, República de Panamá; 4 Instituto de Ciencias Ambientales y Evolutivas, Facultad de Ciencias, Universidad Austral de Chile, Valdivia, Chile; Consiglio Nazionale delle Ricerche (CNR), Italy

## Abstract

*Aplochiton* is a small genus of galaxiid fishes endemic to Patagonia and the Falkland Islands whose taxonomy is insufficiently resolved. Recent genetic analyses confirmed the existence of only two closely related species, *Aplochiton taeniatus* and *Aplochiton zebra*, while a third controversial species, *Aplochiton marinus,* remained lost to synonymy with *A. taeniatus*. Using an integrative taxonomy framework, we studied original samples and published sequences from a broad range in western Patagonia and the Falkland Islands, and generated robust species hypotheses based on single-locus (*Cytochrome Oxidase* subunit *I*; *COI*) species-delineation methods and known diagnostic morphological characters analyzed in a multivariate context. Results revealed three distinct evolutionary lineages that morphologically resemble, in important respects, existing nominal species descriptions. Interestingly, the lineage associated with *A. marinus* was unambiguously identifiable (100% accuracy) both from the genetic and morphological viewpoints. In contrast, the morphology of *A. taeniatus* and *A. zebra* overlapped substantially, mainly due to the high variability of *A. taeniatus*. Discriminant function analysis aided the identification of these species with 83.9% accuracy. Hence, for their unambiguous identification, genetic screening is needed. *A. marinus* has seldom been documented, and when recorded, it has always been found in sites with clear marine influence. It is possible that only *A. marinus* preserves a life cycle related to the sea akin to the hypothesized ancestral galaxiid. We did not find evidence of claimed diadromy in *A. taeniatus* or *A. zebra*, and, therefore, these should be regarded as freshwater species. Finally, a lack of phylogeographic patterns and overrepresentation of uncommon haplotypes suggested demographic expansions in recent evolutionary time, especially of *A. zebra*, in line with the hypothesis of large-scale range expansion and lineage spread in western Patagonia.

## Introduction

Within the galaxiids (Galaxiidae), an austral family of cold-temperate freshwater and putative diadromous fishes, the small genus *Aplochiton* stands out for its phylogenetic distinctiveness, body shape (trout-like), and relatively large body size (360 mm maximum total length) [1]–[3]. *Aplochiton* is endemic to Patagonia and the Falkland Islands [4], and recent accounts suggest shrinking distributions due to the detrimental effects of invasive trout and habitat degradation [5], [6]. Unfortunately, the understanding of species-specific ecological needs and threats of *Aplochiton*, as well as the designation of appropriate conservation statuses have been hampered by poor species delineation and insufficient or misleading knowledge about their distribution and biology [7], [8]. Revising the taxonomy of *Aplochiton* shall enable the implementation of more effective conservation strategies [9].

Three *Aplochiton* species have been described, although one has long been regarded as a junior synonym. Vanhaecke et al. [7], analyzed mitochondrial and nuclear DNA (mtDNA and nDNA, respectively) and published the first genetic description of the group. They confirmed the existence of two closely related species, *A. taeniatus* Jenyns 1842 and *A. zebra* Jenyns 1842 (AT and AZ, respectively). The identification of these species was historically based on morphology, although complicated by high levels of intraspecific variation, and partial character overlap between species [10]–[12]. In fact, meristic and morphometric analyses have not provided clear-cut diagnoses for *Aplochiton* spp. [10]–[12]. Genetic analyses, including mtDNA barcoding, helped identifying AT and AZ and revealed problems associated with traditional morphological identification [7]. For example, morphological misidentification was more widespread and asymmetrical than previously thought – genetically identified AT resembled AZ most of the time (74%) [7]. Furthermore, barcoding allowed the detection of AT where it was previously believed absent, which resulted in the extension of its geographical range to the Falkland Islands [7].

The third *Aplochiton* species, *A. marinus* Eigenmann 1928 (AM), has been rarely recorded, and its taxonomic validity has been questioned ever since its original description [13]. Morphological identification has been tenuous (but see *Results and Discussion: Morphology and ecology*), and, on the basis of apparent allometric growth, specimens of AM have been regarded as possible breeding adults of diadromous (sic) AT, which led to AM been considered a synonym of AT [10]–[12]. Some authors have shown reluctance to accept this synonymisation because the life history and ontogenetic development of the two species are yet to be studied in sufficient detail [5], [14], [15]. Vanhaecke et al. [7] did not find evidence in support of AM, presumably because of the lack of samples for this species.

Herein we adopt the integrative taxonomy framework outlined in Puillandre et al. [16] to test the existence of the three nominal species within *Aplochiton*. Essentially, this framework consists of a four-step methodology that combines traditional taxonomy with modern DNA taxonomy in a workflow optimized to generate robust species inferences [17]–[19]. In particular, (i) *Aplochiton* was sampled in diverse habitat types from a broad geographical range; (ii) primary species hypotheses were proposed based on analyses of the diversity of the mtDNA barcode region (*Cytochrome Oxidase I* gene; *COI*); (iii) genetic affinities were visualized amongst individuals and taxa; and (iv) secondary species hypotheses were consolidated considering additional evidence from nDNA markers [7], morphological analyses, distribution, and ecology.

DNA taxonomy is of central importance in this framework [16], [17], [19]. It provides efficient and generally accurate means for species identification, as well as a theoretical framework for inferential species delineation and discovery [17], [18], [20]. This discipline has evolved substantially since the proposition of DNA barcoding a decade ago [21], [22]. A critical issue has been to objectively delineate species limits based on single-locus mtDNA sequence data. Previous heuristic criteria involved the graphical identification of, or making assumptions about, a barcode gap threshold – or species cutoff point – which was used to differentiate intraspecific from interspecific diversity [21]–[24]. However, generalizations of these criteria have been problematic, especially for closely related species whose barcode gap may be unclear [25]–[27].

For more objective criteria, here we used model-based, single-locus species delineation tools; specifically, the automatic barcode gap discovery method (ABGD) [28], and the general mixed Yule-coalescent method (GYMC) [29], [30]. These methods deliver species circumscriptions based on patterns of pairwise genetic distance (ABGD), or patterns of gene-genealogy branching attributed to either speciation or coalescence processes (GMYC). Because rationales (target criteria) are fundamentally different between these methods, it is advisable to use them in conjunction and challenge each of the resulting primary species hypotheses in light of additional evidence (e.g., nDNA, morphology, and distribution [16]).

DNA taxonomy is especially insightful when morphological identification is tenuous or equivocal [20], [31]–[33]. We showed that this is also the case with *Aplochiton* by attempting the discrimination of species hypotheses on the basis of traditional morphological characters.

Overall, our study reexamined *Aplochiton* diversity from an integrative taxonomy perspective and demonstrated controversial nominal species that have caused much confusion in the systematics and biology of the group. Finally, we discuss misleading knowledge about the ecology of *Aplochiton*, as well as evolutionary perspectives that emerged from our data.

## Results and Discussion

### DNA taxonomy

**Figure 1:**
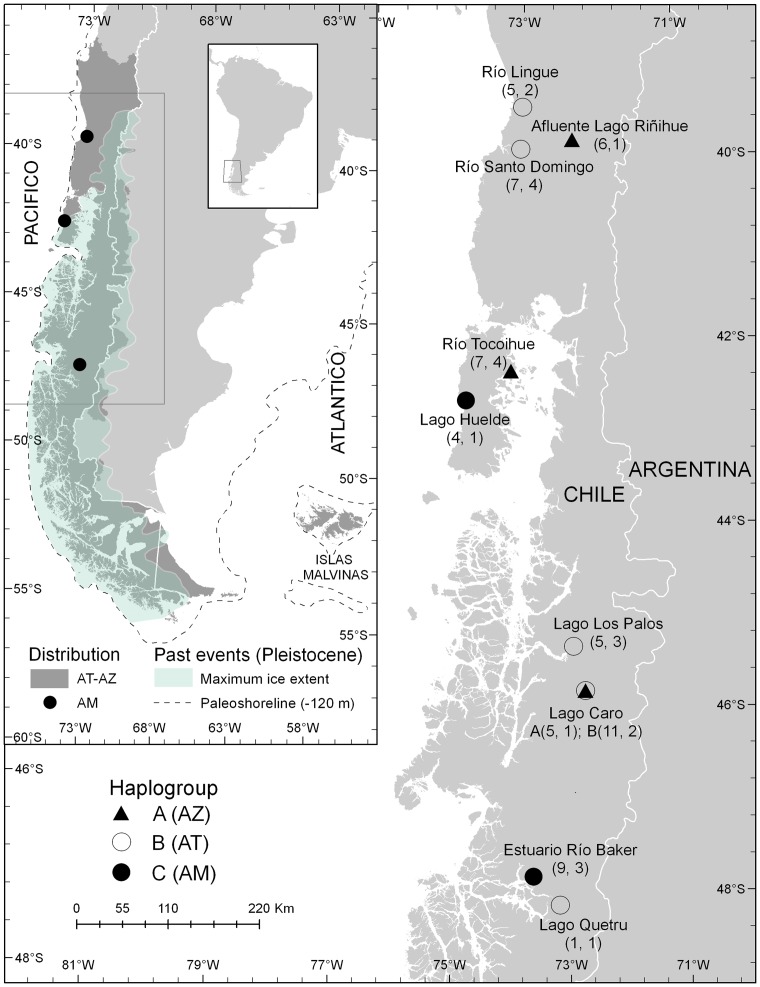
*Aplochiton* distribution range and sampling sites. Distribution ranges of *A. taeniatus* (AT) and *A. zebra* (AZ) have been confused due to equivocal morphological identification, and herein are displayed together (insert, dark area). *A. marinus* (AM) is easier to identify, however, it has been recorded only in a few regions (insert, filled circles). The sampling sites of this study (main map) are indicated with *Cytochrome Oxidase I* (*COI*) haplogroup symbols (legend); in brackets, the sample size followed by haplotype richness. Approximate maximum Patagonian ice sheet extent and shorelines during the Pleistocene were modified from references [82] and [83], respectively.

Our results revealed the existence of three haplogroups within *Aplochiton* highlighting for the first time the high distinctiveness of AM. Specimens were found in rivers, lakes, and estuaries from a broad range in Western Patagonia (Figure 1), and featured substantial morphological variation (see below). We examined the mitochondrial *COI* gene (677 base pairs) of our samples (n = 60), and the published *Aplochiton* haplotypes (n = 10) [7], including haplotypes of *Galaxias platei* (n = 1) and *G. maculatus* (n = 1) as outgroups. The published haplotypes originated from samples collected in North-Western Patagonia (39.6–42.2°S) and the Falkland Islands (in Spanish, Islas Malvinas; 51.5–52.2°S) [7]. These 72 *COI* sequences formed the basis for the construction of primary species hypotheses that were then subjected to further scrutiny considering additional evidence.

**Figure 2:**
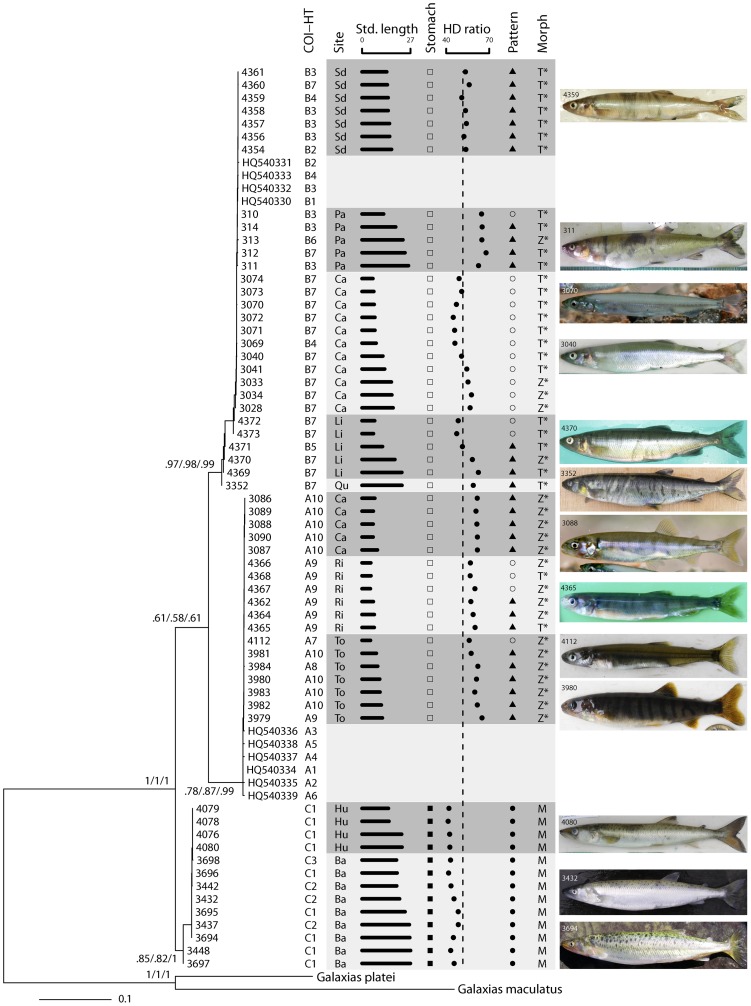
Maximum likelihood tree and three traditional diagnostic morphological characters for *Aplochiton*. Each leaf of the tree is labeled with individual ID-code (this study; n = 60 sequences), haplotype accession number, or outgroup species name (refer to Table 4). Branch support is indicated nearby nodes for inferences based on maximum likelihood (ML, bootstrap), Bayesian Inference (BI, posterior probability), and parsimony (P, bootstrap) (i.e. ML/BI/P); values <0.50 not shown. *Cytochrome Oxidase I* haplotype (*COI*-HT) correspond to those in Vanhaecke *et al.* [7] when matching, or to the new haplotypes described here. The first letters of *COI*-HT stand for haplogroups that were associated with species: A (associated with AZ), B (AT), and C (AM). Additional data are shown with an alternating shaded background to aid the visual separation of each sampling site listed as ''Site'' on the second column (unavailable for sequences downloaded from Genbank). Std. Length is standard length in cm. Stomach displays either the bulbous (□) or elongated (▪) shape. HD ratio is head length to head depth ratio (%), with head depth measured posterior to eye orbit (dashed line corresponds to that in Figure 3-A, for reference). Pattern refers to skin color patterns; dark chevron blotches (▴), dark spots (•) and unclear/no pattern (○). Morph corresponds to morphological identification; letters are initials of the second word (species) in binomial names. Morphological identification was accomplished by reference to Stomach and Pattern (M), or by jackknife identification using linear discriminant analysis based on all five characters analyzed (T and Z; method indicated by *). Note several discrepancies between morphological and barcoding identifications. Some fish photos further illustrate *Aplochiton* phenotypic diversity (IDs correspond tree leaf labels).

Fifty-one polymorphic sites and three *Aplochiton* haplogroups were observed. Relationships in barcode gene diversity were illustrated using phylogenetic trees (gene genealogies) constructed using different analytical methods to assess congruence and robustness [18]. The results of parsimony (P), maximum likelihood (ML) and Bayesian inference (BI) consistently showed three well-supported *Aplochiton* clades (posterior probability (BI) and bootstrap (P, ML) >78% for the three haplogroups; Figure 2). Two clades matched the sequences retrieved from Genbank identified as haplogroups A and B [7], and the third corresponded to a new group identified as haplogroup C. Nucleotide diversity was higher for haplogroup A (π = 0.00206) whereas similar estimates were obtained for haplogroups B and C (π = 0.00095 and π = 0.00085, respectively). The minimum mean (SE) genetic distance (Kimura 2-parameter; K2P) between *Aplochiton* haplogroup pairs was 7.32 (2.03)% observed between A–B (Table 1). Heuristically, *COI* divergences of this magnitude strongly suggest that the observed haplogroups correspond to good biological species [22], [27], [33], [34], although more objective quantitative criteria provided further confirmation.

**Table 1:** Inter- (off-diagonal) and intra-specific (diagonal) pairwise genetic distances.

Haplogroup (Species)	A (AZ); n = 24	B (AT); n = 33	C (AM); n = 13	(GP); n = 1	(GM); n = 1
A (AZ)	**0.0013 (0.0011)**.0.0015 (0.0011)	**0.0732 (0.0203)**	**0.1316 (0.0302)**	**0.1995 (0.0362)**	**0.2766 (0.0426)**
B (AT)	0.0863 (0.0118)	**0.0018 (0.0014)** 0.0019 (0.0014)	**0.1245 (0.0276)**	**0.2298 (0.0386)**	**0.3114 (0.0458)**
C (AM)	0.1095 (0.0108)	0.1009 (0.0103)	**0.0007 (0.0008).**0.0008 (0.0008)	**0.2078 (0.0378)**	**0.2981 (0.0444)**
(GP)	0.2919 (0.0047)	0.2942 (0.0165)	0.2927 (0.0018)	–	**0.2617 (0.0432)**
(GM)	0.3319 (0.0062)	0.3463 (0.0185)	0.3296 (0.0019)	0.2886 (0.0104)	–

Genetic distances (mean (SD)) are K2P distances (boldface) and distances obtained from the best GTR+I+G model (non-bold). Species acronyms as in Figure 1; in addition, *Galaxias platei* (GP) and *Galaxias maculatus* (GM) were included as outgroups.

Further evidence supporting the haplogroup-species correspondence came from quantitative single-locus methods specifically tailored for the delineation of species boundaries. These methods can be divided in two complementary classes: one based on the analysis of pairwise genetic-distance distributions, and the other based on evolutionary models given a genealogic tree topology. For the first class, we used the ABGD method, which estimates a maximum limit for intraspecific genetic divergence and uses this limit to group sequences belonging to the same species (with smaller divergences) from sequences belonging to different species (with higher divergences) [28]. The results showed a multimodal pairwise genetic distance (K2P) distribution with a clear, wide barcode gap located in the range 0.6–6.4% distance (Figure S1a). Furthermore, the method detected three stable candidate species with estimated prior maximum divergence of intraspecific diversity (P) as large as 5.2% (one-tail 95% confidence interval; Figure S1b). Notably, the results matched the three *Aplochiton* haplogroups described above (A, B, and C).

For the second class of methods, we implemented the GMYC. This approach uses pre-defined gene genealogies and implements a model-based analysis to locate threshold points (or nodes) on the genealogy where there are transitions in branching rates reflecting either inter- (speciation) or intra-specific (coalescence) evolutionary processes [29], [30]. Given the estimated transition points, genetic clusters that likely correspond to biological species can be identified. We performed a likelihood implementation of the GMYC model using the maximum clade credibility tree obtained from BEAST and compared models with varying numbers of transition points. Models with single (Likelihood = 73.08939) or multiple transition points (L = 74.68891) were superior to a null model with constant branching rate (L = 65.74721; χ^2^_single_  = 14.6844, df = 2; χ^2^_multiple_  = 17.8834, df = 5; *P*<0.01). Nevertheless, the single threshold model was selected over the multiple threshold model because the latter did not significantly reduce deviance (χ^2^ = 3.19904, df = 3, *P* = 0.3619). Consistent with the ABGD method, the selected single-threshold GMYC model also proposed the same three primary species hypotheses of *Aplochiton* (speciation-coalescent transition, *T* = 0.0855 substitutions per site).

In order to account for uncertainty in genealogy estimation, we also used a Bayesian extension of the GMYC model (bGMYC) [35]. This was performed in a subset of 100 trees sampled from the BEAST's posterior distribution. The results once again clearly corresponded with the three primary species hypotheses or haplogroups (mean speciation-coalescent transition, *T* = 0.0899±0.0086 substitutions per site). Accordingly, posterior probabilities of conspecificity within *Aplochiton* clusters were always high (P>0.89, see Klee diagram in Figure S2).

Next, we challenged each of these mtDNA-based primary species hypotheses (A, B and C hereafter for short) in light of additional evidence. We began by providing additional (existing) evidence in support of A and B as good biological species. It can be misleading to use single-gene approaches to infer evolutionary relationships for example, due to historic events of introgressive hybridization [36], [37]. In this context, congruent patterns between mtDNA and nDNA markers would be valuable evidence in support of diverging phylogenies, particularly of closely related species [16], [18], [34]. Vanhaecke et al. [7] provided just such evidence: they genotyped 367 *Aplochiton* individuals (collected from a broad range) for both mtDNA (*COI* and cytochrome *b*) and nDNA markers (11 microsatellites), and confirmed a congruence between the genetic structure of both mtDNA and nDNA. We are thus confident that at least haplogroups A and B are representative of distinct lineages within the *Aplochiton* phylogeny [7], and, hence, these groups should be promoted to secondary species hypotheses according to Puillandre's framework [16].

Evidence in support of C as a third distinct lineage, as well as links between species hypotheses and nominal species, came from morphological analyses.

### Morphology and ecology

We assessed the usefulness of the most used traditional diagnostic morphological characters, both in a univariate and multivariate context, to differentiate amongst species hypotheses or haplogroups. Haplogroup C was the only species hypothesis featuring unique categorical (but not morphometric) diagnostic characters, namely, dorsal spots and elongated stomach (Figure 2). These unique features are strong evidence that C represents a distinct clade within *Aplochiton*, and, therefore, its status was also upgraded to secondary species hypothesis [16].

It is now opportune to make the link between C and the nominal species AM. Following Eigenmann's [13] original description of AM, and his taxonomic key, individuals of C were identified as AM. Moreover, we examined photographs of AM's holotype (CAS 51274, ex IU 15535), and, aside from obvious differences due to specimen preservation, its general morphology and dorsal spots still visible resembled the individuals we collected. Also there was a clear habitat similarity between C and AM that has always been reported as occurring in locations influenced by the sea (see further discussion below).

**Figure 3:**
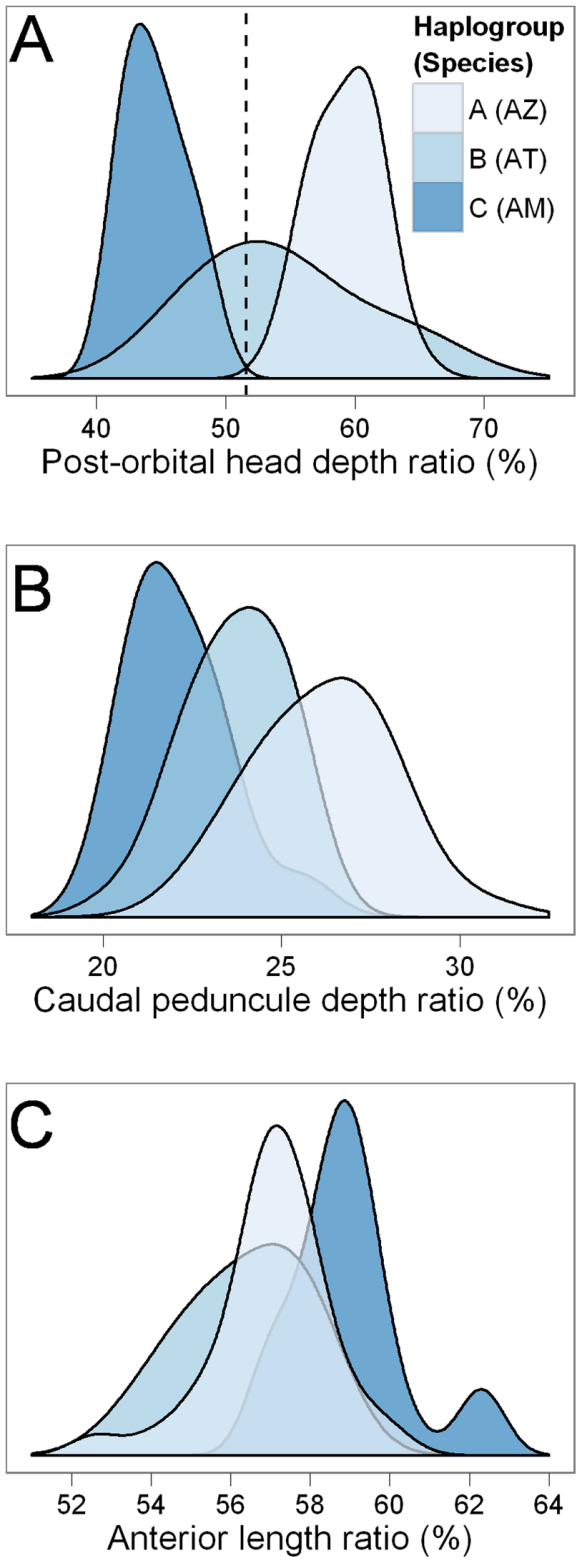
Kernell density distributions by species/haplogroup of the three morphometric characters analyzed (%). (A) Post-orbital head depth to head length ratio. (B) Caudal peduncle depth to standard length ratio. (C) Pre-dorsal length to standard length ratio.

However, linking A and B to nominal species proved more challenging. B featured substantial phenotypic variation and often resembled A both in categorical and morphometric characters (Figure 2). The high phenotypic variability of B was clearly illustrated by the head length to head depth ratio. While C and A were slender- and deep-headed, respectively, B embodied either morph (Figure 3a). Other morphometric characters showed varying degrees of overlap among species (Figure 3b–c).

In spite of these difficulties, we agreed with Vanhaecke et al. [7] that individuals of A should be identified as AZ due to their generally deeper body and chevron bands, while individuals of B should be identified as AT due to their slenderness (e.g., lower head depth ratio) and absence of color patterns in many individuals. These differences are in line with Jenyns' original species descriptions [38]. However, morphological diagnoses are still problematic and future work should focus on the development of field-compatible identification criteria. In the interim, we enhanced the resolution of morphological identification of these species through multivariate discriminant analyses.

**Figure 4:**
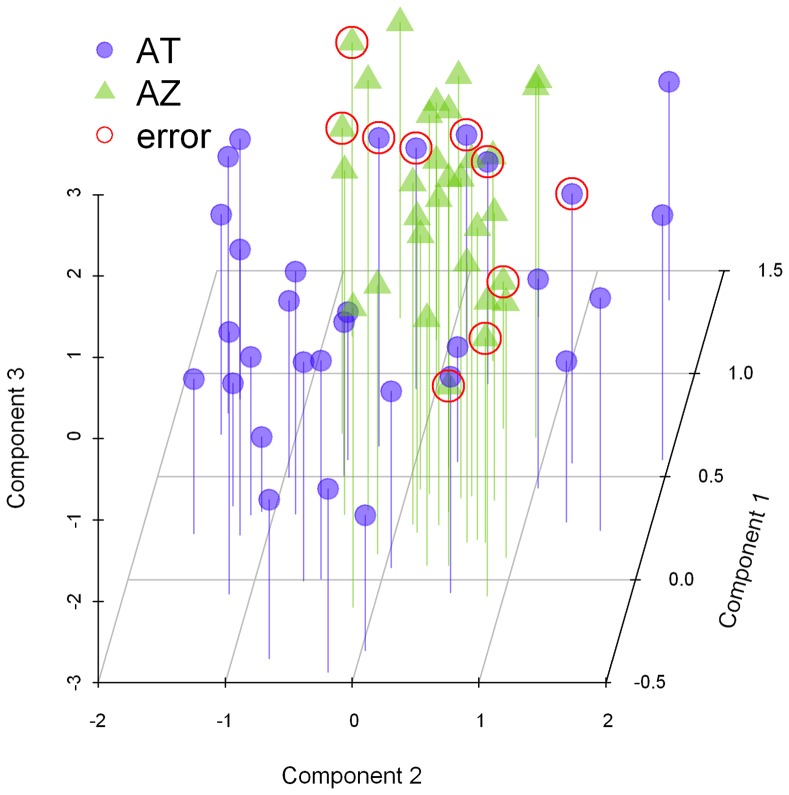
Discriminant space scatter-plot for overlapping morphospecies. Includes 31 AT (•) and 31 AZ (▴) used as training dataset for heteroscedastic linear discriminant function analysis based on four morphological characters. Misidentified cases (16.13%; ○) correspond to incorrect jackknifed predictions.

We conducted smoothed heteroscedastic linear discriminant analysis [39] to assess to what extent it is possible to discriminate AT from AZ on the basis of overlapping categorical (color pattern) and morphometric (those plotted in Figure 3) traditional morphological characters. Species (haplogroup) membership of 52 out of 62 fish (83.9%) was correctly predicted based on morphology, and prediction accuracy was even (symmetric) between AT and AZ (Figure 4; also see identification results in the last column of Figure 2). The discriminative space was dominated by the influence of color pattern (component 1), head depth ratio (component 2), and caudal peduncle depth ratio (component 3) (Table 2).

**Table 2:** Influence of morphological variables on discriminant functions.

Trait	Component 1	Component 2	Component 3
Skin color pattern*	1.20 (**0.70**)	0.00 (**−0.38**)	0.00 (**−0.47**)
Head depth ratio	0.23 (0.06)	0.98 (**0.92**)	0.00 (**0.53**)
Caudal peduncle depth ratio	0.23 (0.07)	−0.42 (0.18)	0.95 (**0.97**)
Pre-dorsal length ratio	−0.25 (0.03)	0.22 (0.06)	−0.24 (0.13)

For each discriminative components obtained from heteroscedastic linear discriminant function analysis, both the loadings of morphological variables and Pearson correlation coefficients are shown (the latter in brackets; significant correlations in boldface). *Dark chevron blotches on fish sides were indexed as 0; unclear/no dark patterns were indexed as 1.

In sum, while it was easy to identify AM based on categorical diagnostic characters (at least the relatively large individuals available), discriminating between AT and AZ was complicated by the high phenotypic variability of AT. In this context, the discriminant function was a useful aid to identify AT and AZ despite morphological overlap (Protocol S1 provides a convenient R application to identify AT and AZ).

Although traditional diagnostic characters were useful to identify species, especially AM, and, to a lesser degree AT and AZ, these same characters had a limited use as originally proposed (e.g., key to *Aplochiton* species identification in reference [12]). For example, individuals featuring the typical AZ morphology with deep head, deep caudal peduncle, and chevron blotches along the body sides [12], can actually be AT (e.g., Figure 2, individuals IDs 311–314). Furthermore, elongated stomach and dorsal spots, which have been related to AT [10], emerged to be unique to AM. AM can also feature (thin) chevron bands, in addition to dorsal spots (CC personal observation), a character that was not apparent on any specimen examined herein. Regardless, individuals of all species can lack clear color patterns, particularly when small, stressed, decaying, or preserved, and hence this character must be interpreted with caution. Further confusion might stem from the rare but possible hybridization between AT and AZ [7]. Therefore, we call for great caution when identifying *Aplochiton* based solely on the use of morphological traits.

Confounded species identification has contributed to misleading knowledge about life history variation. For example, AT and AZ are commonly designated as partially diadromous [2], [4], [10], [12], [40] despite insufficient evidence or based on observations of AM taken as AT. Although individuals of all *Aplochiton* species were collected in environments nearby the sea (particularly Lingue, Tocoihue, Huelde and Baker; Figure 1) only AM was associated to estuarine habitats with strong tidal influence and estuarine by-catch (i.e., Patagonian blennie *Eleginops maclovinus* (Cuvier, 1830)). Furthermore, AM's type locality near Valdivia (40°S) [13], as well as collection sites in the region of Aysén (48°S) of the specimens examined in McDowall and Nakaya [10], [11], are all within or are heavily influenced by the sea. Hence, to our knowledge, there is no evidence of AM ocurring in fully freshwater water bodies, although records are still incomplete. Conversely, AT and AZ usually occur in such environments, sometimes clearly land-locked, and the available literature reveals little or no empirical support for suggested diadromy [2], [4], [10], [40], [41]. Hence, we suggest that only AM preserved a life cycle related to the sea akin to the hypothesized ancestral galaxiid [2]. Detailed studies addressing life history variation and diadromy in *Aplochiton* are lacking. Certainly, further research is needed, for example, analyzing the chemical signatures that are sequentially crystalized in fish otoliths to trace ontogenetic migrations across ecosystems [42], an approach we are already undertaking.

Other misconceptions unveiled by the taxonomic disambiguation of *Aplochiton* refer to the ecology of AT and AZ. We showed that elongated stomach supposedly adapted for fish ingestion actually pertains to AM, not to AT (cf. [10]). Therefore, the strong piscivory suggested for putative AT based on this character [7], [10] lost its support; AT and AZ feature similar, bulbous stomachs suggestive of invertebrate predation [10], [43]. Also, McDowall et al. [44], [45] described the diet and morphological adaptations of putative AZ living in an endorheic, turbid lake on Falkland Islands, yet it turned out that when Vanhaecke et al. [7] barcoded individuals from that same site (Red Pond), they only found AT. This is an example of likely misidentification, and, it is possible that other studies addressing the biogeography, ecology and biology of putative AZ might have inadvertently examined AT [4], [5], [43], [46]–[48]. Future work should critically reframe relevant background knowledge whenever *Aplochiton* species identity matters.

### Evolutionary perspectives

The evolutionary history of *Aplochiton* is virtually unknown. Our goal in this section was to raise some questions and hypotheses from a phylogeographic perspective provided some noteworthy preliminary results. Further confirmation of these hypotheses will require additional research.

*Aplochiton* showed a lack of a geographically structured *COI* gene genealogy and widespread haplotype distributions across a latitudinal range of ∼1,000 Km. This apparent absence of phylogeographic patterns was supported by a relatively low variance among populations compared to the total genetic variance in AM and AT (analyses of molecular variance; AMOVA), and by uncorrelated genetic and geographical distances among populations of the three species/haplogroups (Mantel tests) (Table 3). Although this may not be surprising for highly dispersive marine or diadromous fishes, it is somehow unexpected for freshwater species which commonly show genetic divergence among watersheds [49]. Nevertheless, our methodology was ill-suited for detecting shallow population structure, and at least some structuring was detected in AZ (AMOVA; Table 3). Furthermore, high- resolution clustering based on 13 microsatellite loci [7] also evidenced significant population structure among populations in NW Patagonia (AZ) and in the Falkland Islands (AT), indicating limited contemporary marine connectivity among populations of the ostensibly freshwater species (AT and AZ).

**Table 3:** Genetic variance partitioning (AMOVA), isolation-by-distance (Mantel tests) and neutrality tests (D and F_S_).

Haplogroup (Species)	Percentage of variance (df)			Mantel test	Neutrality tests	
	Haplogroups	Populations	Individuals	r_M_	D	Fs
A (AZ)/B (AT)/C (AM)	97.96 (2) ***	0.88 (7) ***	1.16 (50) ***	−0.26 n.s.	–	–
A (AZ) − 3 pops.	–	61 (2)***	38.52 (15)	0.59 n.s.	−1.88 **	−3.97 ***
B (AT) − 5 pops.	–	19.08 (4)*	80.92 (24)	−0.34 n.s.	−1.55 *	−1.26 n.s.
C (AM) − 2 pops.	–	5.16 (1) n.s.	94.84 (11)	!	−1.15 n.s.	−0.54 n.s.

Levels of significance: >5% (n.s.), 5% (*), 1% (**), and 0.1% (***), but 5% significance level of F_S_ was indicated when P<0.02 [56]. In one occasion, Mantel test could not be conducted due to insufficient number of populations (!). Species acronyms as in Figure 1.

This suggests that the observed phylogeographic patterns (or lack thereof) revealed by our data might represent historic rather than contemporary processes. A plausible scenario involves histories of large-scale extirpations followed by demographic expansions and spread, facilitated by geological and climatic events [50]–[54]. Neutrality tests supported recent demographic expansions, especially for AZ that consistently showed an overrepresentation of uncommon haplotypes, as indicated by statistically significant negative values of both Tajima's [55] D and Fu's [56] F_S_ (Table 3). Although an excess of uncommon haplotypes may provide evidence of a rapid demographic expansion, the potentially confounding effect of genetic hitchhiking and/or purifying selection should be addressed in future research [55], [56].

Demographic expansions during the recent evolutionary history of *Aplochiton* could relate to the increasingly favorable conditions for freshwater fish after peak Pleistocene glacial cycles, as it has been shown for other freshwater biota in Patagonia, including other galaxiids [51], [53], [54], [57]. Species of *Aplochiton* could have retreated to ecological refugia (e.g., northwest) while the Patagonian ice cap stretched over most of their current distribution range (Figure 1, insert). Subsequently, with the melting of glaciers and opening of extraordinary freshwater dispersion routes (e.g., as a result of lowered sea level), refugial lineages could have experienced demographic expansions and spread. The marine affinity of AM could have conferred this species lower vulnerability to the landscape and climatic changes associated with glacial cycles [53]. New molecular studies should include more intensive sampling throughout the distribution of *Aplochiton*, and a wider genome scan in order to test the evolutionary scenarios proposed here.

### Conservation concerns

*Aplochiton* diversity has been underestimated (AM) and confounded (AM, AT and AZ) leading to risky management actions. For example, in Chile, AM has not received conservation status due to its dubious taxonomic validity, whereas AT and AZ are considered in danger of extinction [8]. Furthermore, AM has recently been neglected during the environmental impact assessment of Hidroaysén, the largest hydroelectric project in Chile's history [58]. Many galaxiids use littoral habitat to spawn [1], [47], [59]–[61], and one of the potential impacts of the projected hydropeaking (abrupt caudal changes due to dam operation) is the degradation of galaxiid reproductive habitat. Hence, one of the two AM populations currently known (studied herein) might be at imminent threat. Other factors, such as the geographical expansion of the Chilean salmon industry [62] and the negative impacts of invasive salmonids pose additional and chronic threats to *Aplochiton* conservation [5], [6], [63]. By resolving the taxonomy of the genus this study will inform the decisions of managers responsible for the protection of Patagonian biodiversity.

## Conclusions

Important points emerged by studying the genus *Aplochiton* in more detail, particularly the confirmation of AM as good biological species on the basis of integrative taxonomy. AT and AZ were confirmed as more closely related, and can easily be confused on the basis of traditional morphological criteria. Although multivariate analyses enhanced morphological identification, we echo Vanhaecke *et al.* [7] in that unambiguous identification should resource to DNA analyses until more powerful morphological criteria are developed. Our findings suggested that previously species misidentification might have been widespread in previous studies, and, hence, background knowledge on the biology and ecology of the group must be interpreted critically whenever species identity matters.

## Materials and Methods

### Fish collections

Between 2004 and 2011, *Aplochiton* spp. were collected using various net types from nine locations in a large latitudinal range (39.5–48.1°S) in Western Patagonia, Chile (Figure 1). At each location, fish specimens were euthanized by an overdose of anesthetic solution (tricaine-methanesulfonate or clove oil), placed on a scaled board and photographed (lateral view) using a digital camera. Additionally, a small fin clip was removed and preserved in 95% ethanol for DNA analysis. Voucher specimens were preserved in a 5–10% formalin solution, and a subset was deposited in the Museo Nacional de Historia Natural, Santiago, Chile. A sample of n = 60 individuals representing all localities, different morphotypes and size classes were selected for genetic analysis. The same samples as the genetic analysis, as well as 15 additional individuals (two AT and 13 AZ), were used for multivariate morphological analyses.

Specimens were collected under permits No. 3587, 29 December 2006, and No. 2886, 4 November 2008 (amendment No. 602, 12 February 2009) obtained from the Chilean Subsecretary of Fishing. Our use of and animal handling was approved by the McGill University Animal Care Committee (UACC), Animal Use Protocol No. 5291.

### DNA taxonomy

The fish mitochondrial barcode region was used to identify candidate species or primary species hypotheses. Genomic DNA was extracted from the fin tissue of *Aplochiton* individuals using the EZNA Tissue DNA Kit D3396-02 (Omega Bio-tek, Inc., USA), according to the manufacturer's protocol. We amplified the mitochondrial barcode gene *COI* (677 bp) using the universal primers LCO1490 and HCO2198 and a protocol slightly modified from [64] as follows: PCR was performed in a final volume of 25 μl containing 0.625 units of Taq (MBI Fermentas), 2.5 μl 10X buffer, 3 μl MgCl_2_ (25 mM), 1 μl of each primer (10 pm/μl) [64], 0.5 μl of dNTP mix (10 mM), 1.5 μl BSA (10 mg/ml), 1ul of 1/10 dilutions of DNA extracts, and 13.5 μl of dH_2_O. PCR was performed using an initial denaturation step at 95°C for 5 minutes, followed by 40 cycles of denaturation at 95°C for 45 seconds, annealing at 50°C for 45 seconds, extension at 72°C for 1 minute, and a final extension at 72°C for 10 minutes. PCR products were purified using EZNA Cycle-Pure kit D6493-02 (OMEGA bio-tek) and sequenced using Macrogen custom sequencing service (Macrogen, Seoul, Korea).

The original sequences were edited and aligned using ClustalW multiple alignment option within the software BioEdit [65]. Reading frame errors were checked in the software MACCLADE v. 4.07 [66]. In addition, the *COI* sequences of *A. taeniatus* and *A. zebra* from GenBank were incorporated to the data matrix. Additionally, sequences from two related galaxiids species [2] were also downloaded and used as outgroups (*G. platei* and *G. maculatus*). In total, the data matrix included 72 sequences that were used to conduct the molecular analyses. Table 4 provides GenBank accession numbers of the published and original haplotype sequences analyzed herein.

**Table 4:** GenBank accession numbers for the barcode-region haplotype sequences analyzed.

Haplogroup (Species)	Haplotype	GenBank Accession No.	Reference
A (AZ)	A1 − A6	HQ540334 − HQ540339	[7]
	A7 − A10	KC243102 − KC243104, JQ048551	This study
B (AT)	B1 − B4	HQ540331 − HQ540333	[7]
	B5 − B7	JQ048548, KC243101, JQ048547	This study
C (AM)	C1 − C3	JQ048549, JQ048552, JQ048550	This study
*Galaxias platei*	NA	FJ178349	[84]
*Galaxias maculatus*	NA	AP004104	[85]

Species acronyms as in Figure 1.

Genealogical analyses for the barcode region were conducted using parsimony (P), maximum likelihood (ML) and Bayesian inference (BI). P analysis was performed using the New Technology Search implemented in software TNT, employing a ratchet search method [67], followed by traditional search using TBR branch-swapping, with all characters equally weighted. Nodes support was evaluated by 1,000 bootstrap replicates [68]. ML analyses were performed using RAxML BlackBox (CIPRES Science Gateway website. Available: http://www.phylo.org/sub_sections/portal/. Accessed 2012 October 12) [69]. To model sequence evolution, we employed the GTR+I+G model of nucleotide substitution, which was identified as the best-fitting model based on the Akaike Information Criterion (AIC) using ModelTest v3.8 [70]. Branch support was estimated with 1,000 bootstrap replicates. BI analyses were conducted in BEAST 1.6.2 [71] using the same model of substitution used in our ML analysis. BEAST analysis was run under a strict molecular clock in combination with a Yule speciation process, while all other priors were set to default. In order to check for convergence of the MCMC chains, we performed two independent runs for 80 million generations each, starting from random trees and sampling every 2,000 generations. MCMC output files for the independent runs were pooled together and the parameters of the evolution model were checked in Tracer 1.5 (included in the BEAST package) for effective sample sizes (ESS) >200. A burn-in of 10% was applied once log-likelihood values had stabilised. Maximum clade credibility trees and posterior probability for the nodes were calculated using the last 9,000 sampled trees using TreeAnnotator 1.6.2 (also included in the BEAST package). Genetic distances between different haplogroups were calculated using a K2P and a GTR+I+G distance model using PAUP* 4.0b10 [72].

To objectively delimit species boundaries based on our barcode dataset, we used two quantitative methods that sort sequence information into candidate species. Firstly, we performed ABGD analysis in 'abgd web' (abgd website. Available: http://wwwabi.snv.jussieu.fr/public/abgd/abgdweb.html. Accessed 2012 December 12) selecting the K2P genetic distance [73] and 100 steps (the remaining parameters were set to default). Secondly, we used both a likelihood and a Bayesian implementation of the GMYC model. The former, was performed using the R package ‘splits’ [74] using an ultrametric gene genealogy obtained from our maximum credibility tree from BEAST (see above) [75]. The Bayesian implementation of the GMYC model was performed in the R package ‘bgmyc’ [35] using a subsample of 100 trees from the posterior distribution of BEAST as suggested by the authors. MCMC chains were run for each tree for 10,000 generation with a burn-in comprising the first 1,000 generations once the log-likelihood values had stabilized, and sampling every 100 generations. Repeated haplotypes were removed from both analyses, since identical haplotypes result in zero length branches that could produce an over-partition of the dataset by the model [35].

Finally, we tested for genetic structure by partitioning the genetic variance among haplogroups, among populations within haplogroups, and among individuals within populations (i.e. AMOVA). A series of similar yet reduced analyses was also conducted dividing the dataset by species. Isolation-by-distance was evaluated by Mantel tests using matrices of K2P genetic distances and geographical distances. Departure from neutrality was assessed using Tajima's [55] D and Fu's [56] F_S_ tests. Significant D and F_S_ values can result from selection, bottlenecks, population expansion, or heterogeneity of mutation rates. In particular, F_S_ is a powerful test to detect recent population expansions, which is typically observed as large negative F_S_ values [56]. Significance was evaluated by generating 10,000 coalescent samples. Nucleotide diversity (π), AMOVAs, Mantel tests, and neutrality tests, were conducted in ARLEQUIN 3.5 [76].

### Morphological analysis

In order to test conformity between DNA taxonomy and traditional taxonomy, we mapped traditional diagnostic morphological characters onto the phylogenetic tree, and attempted to identify species based on morphology. In particular, we were interested in learning if traditional diagnostic characters were sufficient to differentiate *COI* haplogroups rather than conducting an exhaustive morphological analysis. We selected two categorical and three morphometric commonly used diagnostic characters for our analysis. The categorical characters were stomach shape and skin color pattern. Stomach shape was examined by dissection and recorded as bulbous or elongated [10]. Skin color patterns were recorded from field observations or photos of fresh fish and coded as chevron blotches, spots, or none/unclear [11], [13], [77].

The morphometric characters were relative head depth, relative caudal peduncle depth, and relative position of dorsal fin. Linear measurements were made on the digital pictures adapting methods from [12]. Head depth was measured close to the occiput [12], as well as at the posterior margin of the fish ocular orbit and at the posterior margin of the operculum, because it was difficult to precisely locate the occiput on fish photos. Since results of pilot analyses were similar when the former two measurements were used, and less clear patterns emerged when the latter measurement was used, we only report the results of the post-orbital head-depth measure. Head depth was expressed as percentage relative to head length, measured from the tip of the snout to the posterior margin of the operculum. Caudal peduncle depth was measured on the narrowest portion of the caudal peduncle, and expressed as percentage of standard length. Distance to dorsal fin or pre-dorsal length was measured from the tip of the snout to the first ray insertion of the dorsal fin, and expressed as percentage of standard length. The density distributions of these characters by haplogroup were visualized by plotting Gaussian kernel densities with bandwidths selected using Silverman's rule of thumb and multiplied by 1.5 for increased smoothness [78].

Because none of these categorical or morphometric characters clearly separated AT from AZ in univariate space, we conducted linear discriminant analysis to maximize separation in multivariate space (AM was clearly identified using categorical, univariate traits and hence was excluded from this analysis). The dataset for this analysis included all 47 barcoded AT and AZ plus 15 additional fish (two AT and 13 AZ) whose identification was deduced by morphology and by assuming they shared the same specific identity as the barcoded fish captured from the same shoal. Thus, the training dataset was balanced with n = 31 AZ and n = 31 AT. We used a heteroscedastic model because within-group covariance matrices on standardized characters differed significantly between species/haplogroups (Permutation test for homogeneity of multivariate dispersions; F1, 60 = 4.7092, *P* = 0.034) [79]. Specifically, we conducted smoothed heteroscedastic linear discriminant analysis [39] as implemented in the ‘hda’ function of the R package ‘hda’ [80]. We specified three discriminative dimensions, and parameters γ and λ were set t o 1 and 0, respectively, following optimization by the ‘train’ function of the R package ‘caret’ [81]. In order to avoid overfitting, we conducted jackknife identification whereby the membership of each individual in the sample was predicted based on a model fitted with that individual excluded from the training dataset (i.e. leave-one-out routine). The accuracy of predictions was then measured as the proportion of correctly identified fish.

## Supporting Information

Figure S1 **Distribution of pairwise distances for the** ***COI*** **barcode gene and automatic barcode gap discovery (ABGD) results.** (A) Frequency distribution of K2P distances between haplotype pairs for the *COI* barcode gene. (B) ABGD results showing the number of groups obtained for a range of prior maximum divergence of intraspecific diversity. Dashed lines (A and B) indicate the upper bound of estimated maximum limits for intraspecific genetic divergence that resulted in three stable candidate species.
(TIFF)

Figure S2 **Species delineation based on GMYC and bGMYC.** The cladogram is the maximum clade credibility tree obtained from BEAST. Clades highlighted in red represent the maximum likelihood species limits from GMYC analysis. Results from the bGMYC method are presented in a haplotype-by-haplotype matrix where cells are color-coded based on the posterior probability of conspecificity between the assorted haplotype pairs.
(TIF)

Protocol S1 **R script to identify AT and AZ based on morphology.** The script loads the heteroscedastic linear discriminant function described in this article (file HLDF.RData), reads a table with new morphological data provided by the user (the original data is provided as an example; file MorphoData.csv), and produces summary results, graphics, and a table with the resulting identification.
(RAR)
