# Supplementary figures and images for: Diversity of Aplochiton Fishes (Galaxiidea) and the Taxonomic Resurrection of A. marinus
Source: PLoS One. 2013 Aug 19;8(8):e71577. doi: 10.1371/journal.pone.0071577 (PMC3747208; doi:10.1371/journal.pone.0071577)

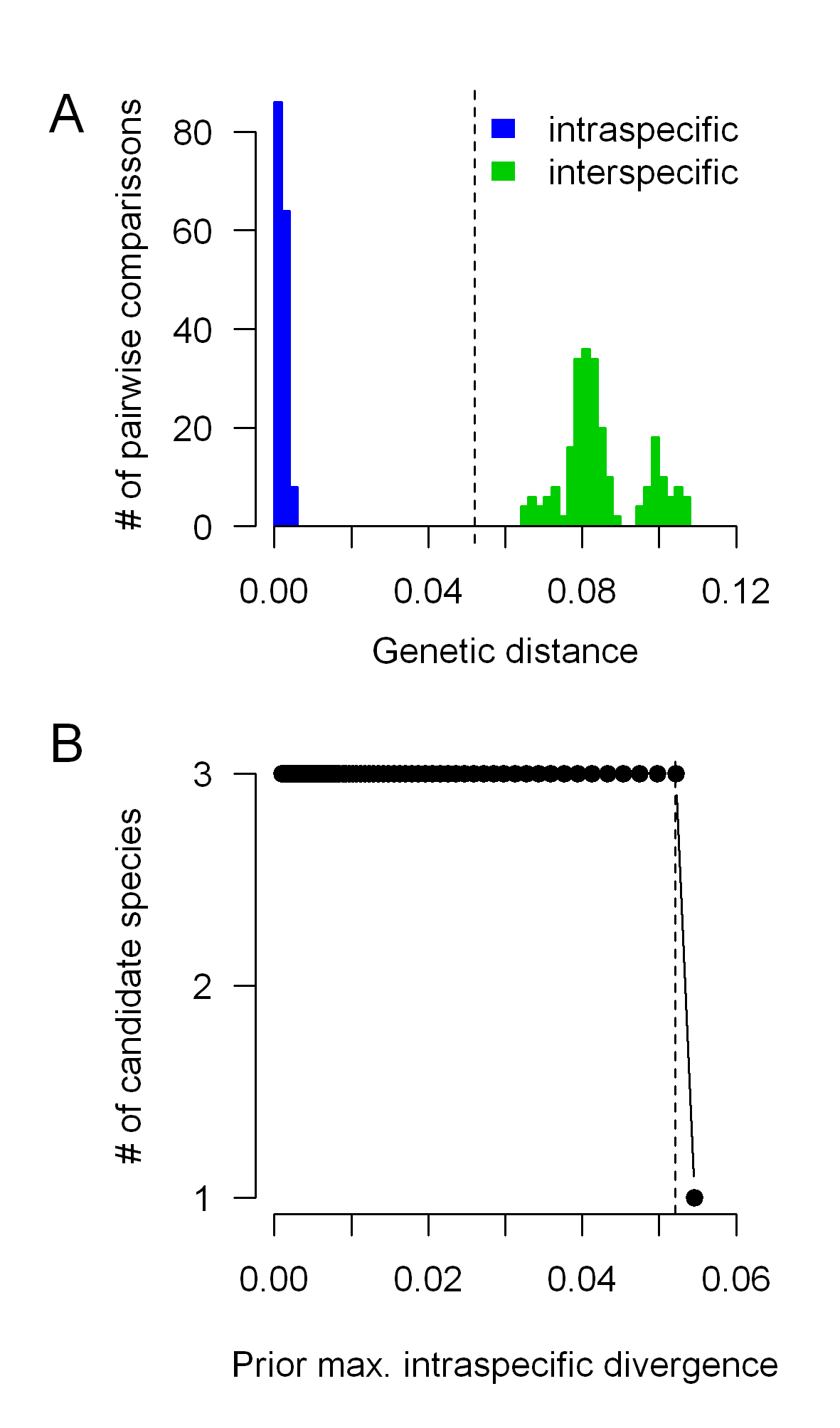

Supplement: Figure S1 — Distribution of pairwise distances for the COI barcode gene and automatic barcode gap discovery (ABGD) results. (A) Frequency distribution of K2P distances between haplotype pairs for the COI barcode gene. (B) ABGD results showing the number of groups obtained for a range of prior maximum divergence of intraspecific diversity. Dashed lines (A and B) indicate the upper bound of estimated maximum limits for intraspecific genetic divergence that resulted in three stable candidate species. (TIFF) [file pone.0071577.s001.tiff]

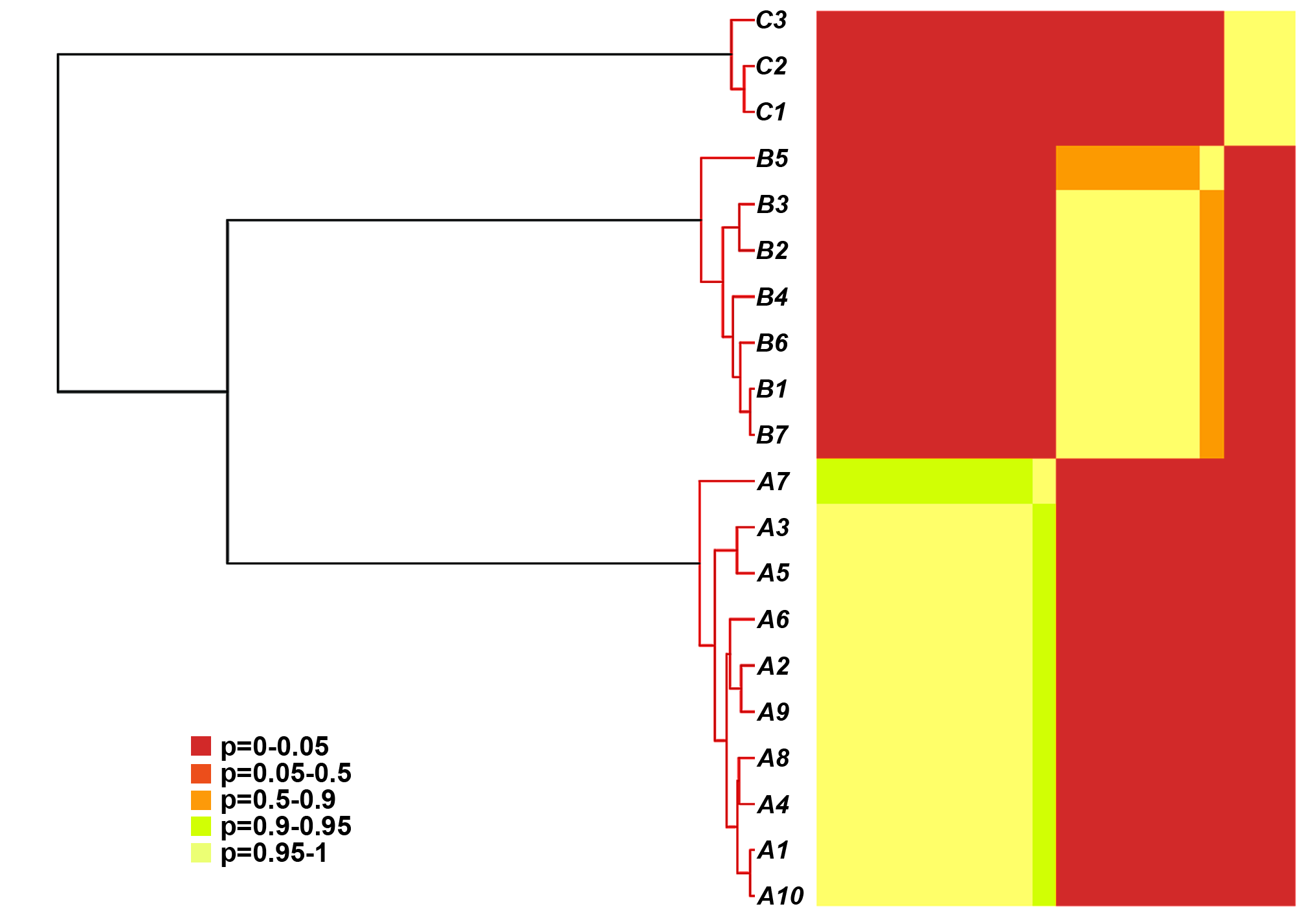

Supplement: Figure S2 — Species delineation based on GMYC and bGMYC. The cladogram is the maximum clade credibility tree obtained from BEAST. Clades highlighted in red represent the maximum likelihood species limits from GMYC analysis. Results from the bGMYC method are presented in a haplotype-by-haplotype matrix where cells are color-coded based on the posterior probability of conspecificity between the assorted haplotype pairs. (TIF) [file pone.0071577.s002.tif]
